# Temperature, Disinfectant Concentration and Bacterial Innate Properties Influence the Disinfection of Bacterial Pathogens Relevant to Catfish Aquaculture

**DOI:** 10.1111/jfd.70216

**Published:** 2026-05-30

**Authors:** Aloysius Chimezie Adibe, Margaret Crumlish

**Affiliations:** ^1^ Institute of Aquaculture University of Stirling Stirling UK; ^2^ Department of Fisheries and Aquaculture Nnamdi Azikiwe University Awka Awka Anambra State Nigeria

**Keywords:** aquaculture biosecurity, aquaculture disinfectants, aquatic pathogen control, broth microdilution suspension test, carrier test method, MIC/MBC determination, Virkon aquatic disinfectant

## Abstract

Aquaculture systems suffer a wide range of losses, mostly due to bacterial disease outbreaks, and the use of disinfectants is a routine practice for controlling the spread of pathogenic organisms globally. Including *Aeromonas hydrophila* , *Streptococcus agalactiae* and *Edwardsiella tarda* , implicated for natural disease outbreaks and economic losses in catfish culture systems. Unfortunately, suboptimal disinfection practices are widespread within catfish aquaculture systems, and information on the factors influencing disinfection efficacy is limited. In this study, the efficacy of Virkon aquatic disinfectant against pathogenic strains of *A. hydrophila* , *S. agalactiae* and *E. tarda* was investigated by measuring changes in absorbance and viable colony counts at 22°C, 28°C and 34°C. The in vitro assays were conducted via broth microdilution suspension and carrier test methods to investigate the influence of disinfectant concentration, strain variation and temperature fluctuations on disinfection efficacy within catfish aquaculture systems. The results revealed that 0.125% was the MIC and MBC for the *S. agalactiae* strains under all the experimental conditions in both tests. However, at 28°C and 34°C, the MIC and MBC were 0.25% for *A. hydrophila* strains in the suspension test and 0.5% for the carrier test. Among all the strains, *E. tarda* presented the highest level of resistance to the disinfectant, with 1% MBC in the carrier test at 22°C and 28°C. In addition, the MIC value for strains of *A. hydrophila* and *E. tarda* was 0.25% at 28°C and 34°C in the suspension test, and both species had the same MBCs of 0.25% at 28°C in the suspension test and 0.5% at 34°C in the carrier test. Overall, changes in temperature, disinfectant concentration and intrinsic bacterial factors were identified as the principal factors that influenced the disinfection efficacy of Virkon aquatic disinfectant. Our findings identified suboptimal sanitation practices as high aquaculture biosecurity risk and emphasized that the carrier test is an ideal method for evaluating the efficacy of chemical disinfectants applied in the context of aquaculture.

## 1 Introduction

Disinfection is an important part of biosecurity strategies and, as such, is widely applied in aquaculture to control the entry, transmission and exit of pathogenic organisms within farming systems and shared water bodies (Othman et al. 2024). Infectious disease outbreaks constantly threaten the sustainability of aquaculture systems globally and consequently cause enormous economic losses (Assefa and Abunna 2018). In freshwater farming systems, infectious diseases caused by pathogenic bacteria, including *Aeromonas hydrophila*, *Edwardsiella tarda* and *Streptococcus agalactiae*, have been associated with natural disease outbreaks within aquaculture systems worldwide (Iregui et al. 2016; Jiang et al. 2016; Semwal et al. 2023). Although these organisms are not considered obligate pathogens, they continue to cause infectious diseases within a diverse range of freshwater fish species and farming systems globally. The motile *A. hydrophila* , the causative agent of motile Aeromonas septicaemia (MAS), which has an optimal temperature ranging from 22°C to 37°C (Semwal et al. 2023), often causes high mortality in freshwater aquaculture systems globally (Bhattacharya Sanyal et al. 2018; Irshath et al. 2023; Rashid et al. 2014). In contrast, *E. tarda* survives over a wide temperature range, ranging from 14°C to 45°C (CABI Compendium 2019), and can infect both seawater and freshwater fishes (Li et al. 2022; Xu and Zhang 2014). However, *S. agalactiae* survives at temperatures ranging from 20°C to 35°C (Laith et al. 2017) and causes disease outbreaks in multiple fish species (Deng et al. 2017; Iregui et al. 2016; Mugetti et al. 2022; Pierezan et al. 2020), that is recognized as *streptococcosis* (Iregui et al. 2016).

Disinfectants are chemical agents (Maillard and Pascoe 2024; Wales et al. 2021) that can interrupt the cell wall and cell membrane development in microorganisms (Mon‐on et al. 2018; Tong et al. 2021). They are often considered to have a broad spectrum or nonspecific nature. In other words, they target a diverse range of pathogens and are described as bactericidal, sporicidal, fungicidal, virucidal or mixed (Ortega Morente et al. 2013). These substances have been used in food production, human medicine and veterinary medicine since the early 20^th^ century (Bedoya et al. 2021; Ortega Morente et al. 2013; Östman et al. 2017). However, only a few iodophor compounds, quaternary ammonium compounds (QACs) and glutaraldehyde‐based disinfectants are commonly used to control the spread of infectious pathogens in aquaculture systems globally (Mon‐on et al. 2018). However, glutaraldehyde‐based disinfectants are discouraged in aquaculture because of their reported toxicity to aquatic organisms, including fish, even at low concentrations (Pereira et al. 2014). Presently, many QACs, iodophor, isopropyl alcohol, phenol, chlorine and ethyl alcohol‐based disinfectants are used in aquaculture for surface cleaning and sanitation purposes (Bowker et al. 2019; Mohamed Din and Subasinghe 2024; Mon‐on et al. 2018). Among several branded disinfectants licensed for aquaculture use, Virkon aquatic disinfectant has wide global acceptance in both small and large facilities (Stockton‐Fiti and Moffitt 2017). Owing to its broad‐spectrum efficacy against various fish viruses, fungi and bacteria at 1% and 0.5% concentrations (LANXESS 2025; Mohamed Din and Subasinghe 2024). Virkon aquatic disinfectants possess multiple modes of action against several pathogenic cells. Particularly, they oxidize sulphur bonds in proteins and enzymes and rupture the cell wall, thereby altering the cell membrane's function and leading to eventual inactivation (Acosta et al. 2021).

Nevertheless, the disinfection efficacy of chemical disinfectants depends on the interplay of multiple factors, including the innate properties of target microorganisms as well as human and environmental factors (Wales et al. 2021). The most common human errors include improper disinfectant preparation, the use of suboptimal disinfectant concentrations, inappropriate disinfectant storage, variations in temperature and pH, and elevated levels of organic matter in the water (Chowdhury et al. 2019; Gongora et al. 2024). These factors influence the contact time of disinfectants with microorganisms, leading to poor efficacy. Furthermore, the influence of microbial strain variation and virulence factors, including motility, lipopolysaccharides (LPS), biofilm formation and spore production, also impacts proper disinfection (Mon‐on et al. 2018). Overall, temperature variation, disinfectant type, concentration and contact time are frequently investigated. Whereas the influence of bacterial innate properties on disinfection efficacy against aquatic bacterial pathogens has been understudied (Piskovsky and Oliveira 2023; Zhao et al. 2023).

Chemical disinfectants are assessed to ensure safety, efficacy and quality control prior to marketing, and their application as part of biosecurity measures requires evidence‐based data on efficacy (Wales et al. 2021). In most cases, single tests or combinations of tests, including suspension, carrier, capacity, surface and field tests, are used to evaluate the disinfection efficacy of several chemical disinfectants (Ecolab 2020; Wales et al. 2021). Among these tests, both suspension and carrier tests are commonly used in laboratory‐based assays because they are robust and adaptable to different laboratory settings and conditions (Beckloff et al. 2007; CLSI 2018). Interestingly, the literature on the efficacy of hygiene, sanitation and disinfection for fish pathogens is large and spans several decades. Table 1 provides an overview of the disinfection type, pathogens and limiting factors affecting disinfection efficacy for common bacterial pathogens and diseases in global aquaculture.

**TABLE 1:** Previous studies on disinfection efficacy tests against fish pathogens.

Disinfectants	Organism investigated	Factors considered	References
Povidone iodine, QACs, Glutaraldehyde	*S. agalactiae*	Contact time, temperature, soiling, disinfectant type, concentration.	Mon‐on et al. (2018)
Virkon aquatic, hydrogen peroxide, peracetic acid	*Vibrio anguillarum, V* *. harveyi* , *V. alginolyticus, Photobacterium damselae subsp. Damselae*	Concentration, biofilm formation, strain variation.	Acosta et al. (2021)
Hydrogen peroxide	*Escherichia coli, Staphylococcus aureus, Enterococcus hirae, Pseudomonas aeruginosa*	Disinfectant type, concentration, strain variation.	Ríos‐Castillo et al. (2017)
Accelerated hydrogen peroxide, QACs, sodium hypochlorite	*S. aureus, P. aeruginosa*	Strain variation, disinfectant type, concentration, contact time.	West et al. (2018)
Citric acid, QACs, potassium peroxymonosulfate, sodium dichloroisocyanurate, glutaraldehyde	*Salmonella enterica* serovar Typhimurium	Contact time, temperature, disinfectant type.	Jang et al. (2017)
Virkon‐S, biosentry iodine, hydrogen peroxide	*A. hydrophila, V. vulnificus*	Contact time, disinfectant type, strain	Eman et al. (2015)

The application of disinfection as an integral part of biosecurity measures is widespread in developed aquaculture sectors. However, little information is available regarding disinfection practices within catfish aquaculture systems, especially in developing countries, including Nigeria, a country with high intensification and the largest producer of African catfish ( *Clarias gariepinus* ) (FishStat 2024). Hence, to provide supportive information for enhancing sanitation practices as part of aquaculture biosecurity, this study investigated the influence of temperature fluctuations, disinfectant concentration and bacterial innate properties on disinfection efficacy within catfish aquaculture systems by measuring changes in absorbance and viable colony counts.

## 2 Materials and Methods

### 2.1 Bacterial Isolates, Inoculum Preparation and Confirmatory Identification Tests

A total of 5 bacterial isolates reported to cause infectious disease outbreaks within the catfish species were investigated (Table 2). All the isolates were provided by Prof. M. Crumlish from bacterial stocks previously identified to the species level prior to storage at −70°C. The *A. hydrophila* strains were recovered from the kidneys of moribund striped catfish (*Pangasianodon hypophthalmus*) farmed in earthen ponds. Whereas strains of *S. agalactiae* strain 6 were isolated from kidneys of clinically sick Nile tilapia ( *Oreochromis niloticus* ) farmed in river‐based cages. Both *E. tarda* NCIMB 2034 and *S. agalactiae* NCIMB 701348 were purchased from a commercial culture collection. Prior to storage, molecular identification via *the rpoD* gene confirmed *A. hydrophila* strain 15 to be *A. dhakensis* and strain 97 to be *A. hydrophila* . Prior to isolate use, confirmatory identification tests, such as motility, oxidase, oxidation‐fermentation and Gram staining, as detailed in Table 2, were performed according to established methods (Crumlish et al. 2002; Waltman et al. 1986). All the bacterial isolates were recovered on Tryptone Soy Agar (TSA) (E&O Laboratory, UK) from Protect Beads (SWA1147; SLS, UK) stored at −80°C. The bacterial concentration in cfu/mL was confirmed via the drop count method (Miles et al. 1938).

**TABLE 2:** Properties and concentrations of bacterial isolates used in the study.

Bacterial isolates	Properties					Initial concentrations in test (cfu/mL)		
	Motility	Gram stain	Shape	Oxidase	O/F	OD_600nm_	Carrier	Suspension
*A. hydrophila* strain 15	Motile	−ve	Rod	+ve	Fermentative	0.99	3.89 × 10^8^	1.94 × 10^7^
*A. hydrophila* strain 97	Motile	−ve	Rod	+ve	Fermentative	1.01	2.55 × 10^8^	1.27 × 10^7^
*E. tarda NCIMB* 2034	Motile	−ve	Rod	−ve	Fermentative	1.02	4.70 × 10^8^	2.35 × 10^7^
*S. agalactiae* strain 6	Non motile	+ve	Cocci	−ve	Oxidative	1.01	4.33 × 10^8^	2.16 × 10^7^
*S. agalactiae NCIMB* 701348	Non motile	+ve	Cocci	−ve	Oxidative	0.97	2.33 × 10^8^	1.16 × 10^7^

*Note:* The table shows the species of bacteria, strains, properties and concentrations: −ve, negative; +ve, positive; O/F, oxidative–fermentative; OD, optical density.

### 2.2 The Test Disinfectant and Preparation

Virkon aquatic powder (LANXESS, Germany) was collected from stock disinfectants used at the Institute of Aquaculture (IoA), UoS. Then stored in airtight containers at room temperature. The product contains potassium peroxymonosulfate, sodium dodecylbenzene sulfonate, sulfamic acid and inorganic buffers as active chemical agents. The disinfectant was freshly prepared for each test following the manufacturer's instructions and was used within 5 h after preparation. A 30 mL volume of 2% concentrated working stock was made by dissolving 60 g of disinfectant in distilled water. Subsequently, the samples were serially diluted in Tryptic Soy Broth (TBS) (E&O Laboratory, UK) to obtain 1%, 0.5%, 0.25% and 0.125% disinfectant concentrations.

#### 2.2.1 Broth Microdilution Suspension Test

The suspension test was conducted using the broth microdilution method described by (CLSI 2018) and (Beckloff et al. 2007), with modifications. In detail, 50 μL of sterile TSB was aseptically added to 4 columns of sterile flat bottom 96‐well plates (VWR, China) in triplicate. Then, 50 μL of disinfectant was added to the first column, and 2‐fold serial dilutions were performed to obtain four different concentrations: 1%, 0.5%, 0.25% and 0.125% disinfectant. A bacteria control well was prepared for each row with 50 μL of sterile TSB only, and 3 sterility control wells were prepared with 100 μL of sterile TSB only. Then, 50 μL of bacterial suspension was added to the various test and bacteria control wells. Subsequently, mixed thoroughly by pipetting to achieve a final bacterial concentration of 10^7^ cfu/mL (Table 2). At the end of preparation, 4 test groups and 2 control groups were established with 3 replicates per assay: test group 1 (bacteria +1% disinfectant), test group 2 (bacteria +0.5% disinfectant), test group 3 (bacteria +0.25% disinfectant), test group 4 (bacteria +0.125% disinfectant), a bacteria control (bacteria + TSB) and a sterility control (TSB only), as shown in Figure 1.

**FIGURE 1:**
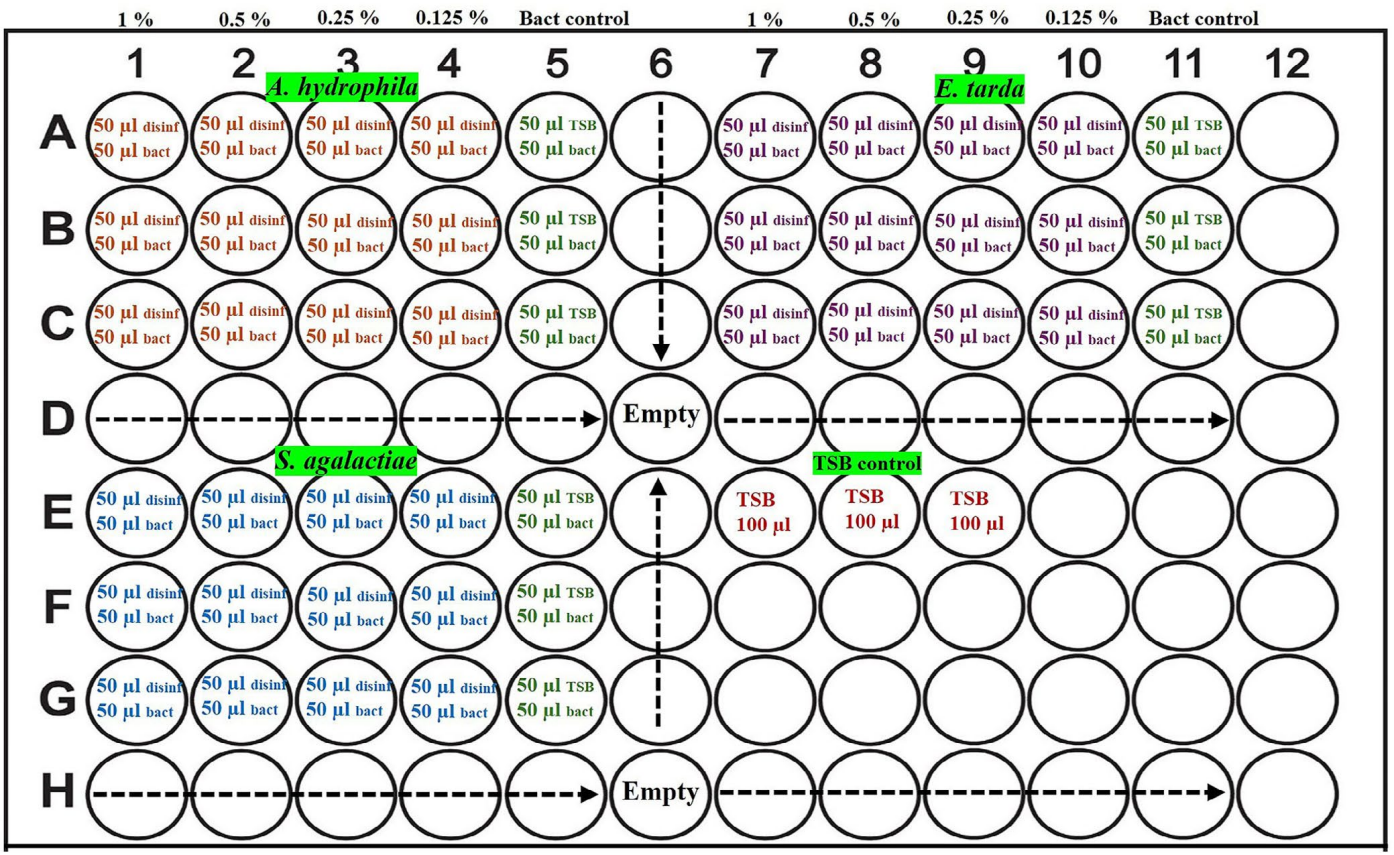
Experimental layout for broth microdilution suspension test. Layout of the experimental design for the microdilution suspension test assay performed in 96‐well plates with strains of *A. hydrophila, E. tarda and S. agalactiae*. bact, bacteria; Disinf, disinfectant; TSB, Tryptic Soy Broth.

Then, the 96‐well plate was sealed with film (Starlab, USA), and the pre‐incubation absorbance (A600_nm_) was measured with a spectrophotometer (Bio Tek, UK). The readings were taken twice at 20 s intervals at 28°C, with continuous agitation between readings. This was to promote thorough mixing and ensure contact between the disinfectant and bacterial cells. The plate was then covered with its lid, sealed and subsequently incubated at 22°C, 28°C and 34°C for 24 h at 100 rpm in an orbital incubator (Kuhner, Switzerland). The test temperature conditions were selected in relation to optimal growth conditions of examined pathogens and the average temperature range in Nigeria (Danba et al. 2015; Emem et al. 2024).

#### 2.2.2 Minimum Inhibitory Concentration (MIC) Determination

The MIC, described as the lowest concentration of a disinfectant that prevents visible turbidity after 24 h of incubation (CLSI 2018), was determined for each bacterial isolate. After 24 h of incubation, the 96‐well plates were removed from the incubator, followed by absorbance reading as described above. The test groups and sterility control wells were grossly observed for turbidity by placing the plate on a dark‐non‐reflecting surface, and the test group, which had no visible turbidity compared with the bacteria control well, was selected as the MIC. Additionally, pre‐ and post‐incubation absorbance values for each test group were expressed as the mean values and compared with the absorbance values of the bacteria control well (CLSI 2018).

### 2.3 Carrier Test

Carrier tests were conducted to determine the disinfection efficacy of nets commonly used in hatchery and fish production facilities. A fine mesh net that was used at the IoA tropical aquarium was provided by Mr. B. Craig. The net was cut approximately 2 × 2 cm and sterilized in an autoclave (Austell, UK). The test was performed as described previously (Jang et al. 2017), with modifications. In detail, a sterile net was soaked for 15 min in a bacterial suspension at 10^8^ cfu/mL. Four test groups were prepared via 2‐fold serial dilution with 2% disinfectant in sterile TSB in a 4 mL total volume to obtain 1%, 0.5%, 0.25% and 0.125% disinfectant concentrations as detailed in Table 2. Sterility and bacteria control groups were also prepared with 4 mL of sterile TSB. All assays were conducted in duplicates with 30 mL sterile universal containers. After 15 min, the net was aseptically inoculated into the respective test groups and bacteria control groups. For the sterility control groups, a sterile net not exposed to bacteria was added for the net sterility control, whereas the TSB sterility control contained only sterile TSB as described in the experimental layout in Table 3. The containers were subsequently incubated appropriately at 22°C, 28°C and 34°C for 30 min (Wales et al. 2021). After 30 min of contact, the nets were aseptically transferred into 10 mL of TSB in another set of sterile containers for bacterial recovery and incubated at 28°C for 24 h for *A. hydrophila* and *E. tarda* strains and 43 h for *S. agalactiae* strains as shown in Figure 2. The *S. agalactiae* strains were incubated longer because pilot test revealed that they required more time to regrow after treatment with the disinfectant. After 24 or 43 h of incubation, the optical density (OD_600nm_) of the solution in each container was measured with a cell density metre (Biochrom, UK), and bacterial growth was quantified by viable colony counts (Miles et al. 1938).

**TABLE 3:** Experimental layout for the carrier test.

Treatment groups	Description	Content
Test group 1	Disinfectant + bacteria	1% disinfectant + bacteria
Test group 2	Disinfectant + bacteria	0.5% disinfectant + bacteria
Test group 3	Disinfectant + bacteria	0.25% disinfectant + bacteria
Test group 4	Disinfectant + bacteria	0.0125% disinfectant + bacteria
Control group 1	Bacteria control	Bacteria + sterile TSB
Control group 2	Net sterility control	TSB + sterile net
Control group 3	TSB sterility control	Sterile TSB only

**FIGURE 2:**
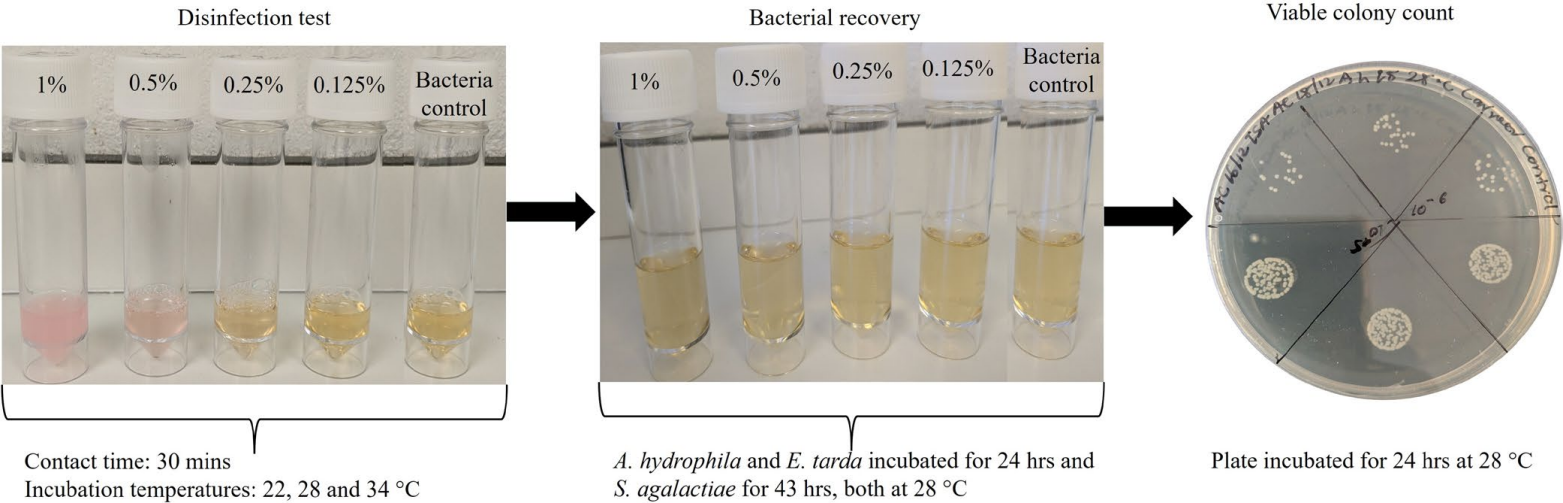
Carrier test experimental layout. Illustration of steps followed in the carrier test.

### 2.4 Minimum Bactericidal Concentration (MBC) Determination

The MBC of Virkon aquatic disinfectant was determined in both tests as described by (CLSI 2018). For the suspension tests, the wells corresponding to the MIC, along with the adjacent wells immediately above and below the MIC, were selected for further analysis. The contents of these wells were aseptically transferred into 10 mL of sterile saline solution. This dilution step was performed to stop the bactericidal activity of the disinfectant. The selected wells were washed twice to ensure complete recovery of the bacterial cells. Subsequently, to determine the MBC, 20 μL of suspension was aseptically dropped on TSA plates in triplicate.

Similarly, for carrier tests, containers that showed no visible turbidity, along with the adjacent groups immediately above and below the container, were selected from the bacterial recovery containers. Subsequently, 20 μL of the suspension from each container was plated on TSA plates in triplicate. Additionally, the bacteria control and sterility control plates were prepared in an equivalent manner using the corresponding control groups. In both the suspension and carrier tests, prior to plating, a 10–fold serial dilution was performed for test groups that exhibited high turbidity. This step ensured that the resulting bacterial concentrations fell within a countable range, allowing accurate quantification of viable colonies on the TSA plates.

After plating, all the plates were sealed, incubated at 28°C and monitored for 24–48 h. The MBC for each bacterial strain was determined as the lowest disinfectant concentration that did not permit visible bacterial growth on the surface of the TSA plate after 48 h of incubation (CLSI 2018). Viable colony counts were conducted (Toribio‐Avedillo et al. 2020), and total recovered bacteria were recorded for each test group and compared to the bacteria control. Oxidase and Gram tests were conducted to confirm the identities of the recovered bacteria. Additionally, the physical appearance of colonies recovered from the test groups was compared to that of the bacteria control group to confirm uniformity or otherwise. All the assays were confirmed by positive bacterial growth in the bacteria control group and no bacterial recovery from the sterility control groups.

### 2.5 Data Analysis

Viable bacterial colony recovery data from suspension and carrier tests and optical density data from carrier tests were expressed as the mean values. The absorbance data from the suspension test were expressed as the means and standard deviations and were evaluated for normality before analysis of variance (ANOVA) was conducted. Tukey's HSD (honestly significant difference) test was subsequently used to determine the statistical significance of differences in absorbance readings within treatment groups, incubation periods and between treatment groups and incubation periods. All statistical analysis was conducted in JMP software (version 18.0.1) and *p* values < 0.05 were considered to indicate statistical significance between the compared groups.

## 3 Results

### 3.1 Broth Microdilution Suspension Test

In vitro suspension tests were conducted at microvolumes to determine the inhibitory and bactericidal concentrations of the test disinfectant against the test bacterial strains. In all the assays performed, the natural pink colour of the Virkon Aquatic disinfectant resulted in high absorbance values at a 1% concentration, but the absorbance decreased at lower concentrations after serial dilutions. The absorbance values of the test and bacteria control groups were normalized by subtracting the readings in the empty well, which served as a negative control. For all the isolates, ANOVA indicated that the means of the absorbance readings differed significantly among the treatment groups across the incubation temperatures and between the treatment groups and incubation temperatures (*p* < 0.0001).

#### 3.1.1 *Aeromonas hydrophila* Strain 15

According to the absorbance records, OD values at 0 h were lower than 0.2 for all the test groups, regardless of incubation temperatures (Figure 3A). The values remained low (< 0.2) for the 1% (dark blue), 0.5% (orange) and 0.25% (green) test groups after 24 h incubation at 28°C and 34°C. Whereas the 0.125% group (light blue) showed increase (Figure 3A). Additionally, this rise in OD for the 0.125% group was significantly higher than that in the control group at 34°C (*p* = 0.0196), 22°C (*p* < 0.0001) and 28°C (*p* = 0.0004). These findings suggest that the lower concentration of disinfectant is ineffective under higher temperatures. Furthermore, changes in OD values at 24 h were correlated with viable bacterial recovery to confirm the bactericidal activity of the disinfectant. No bacterial colonies grew in the higher concentrations under any of the test temperatures, but the 0.125% group had viable bacteria at 28°C and 34°C (Figure 3B). Comparably, the number of bacterial recovered from the 0.125% group at 28°C was greater than the initial bacterial concentration, whereas an over 5–log reduction was recorded at 22°C (Figure 3B).

**FIGURE 3:**
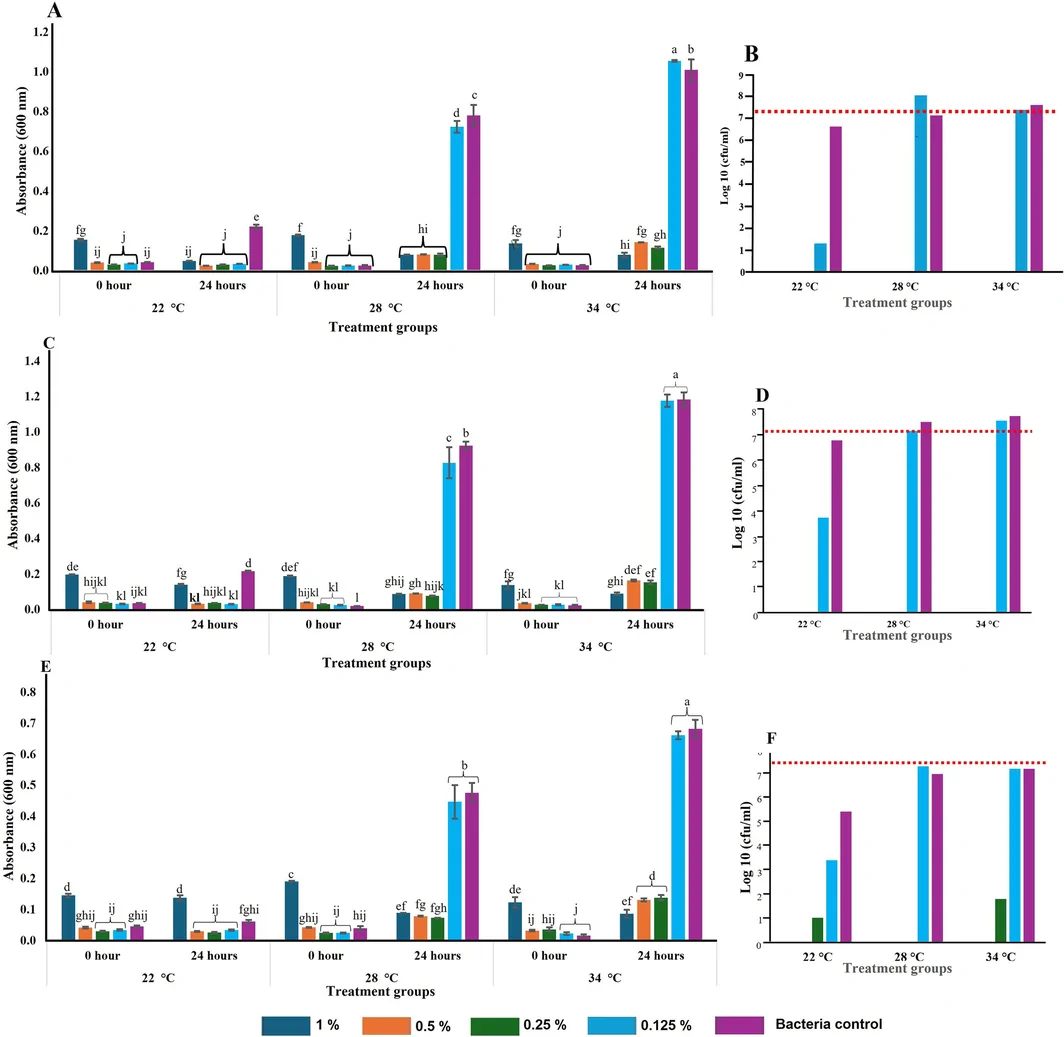
Absorbance and viable colony count of *A. hydrophila* and *E. tarda* isolates. The mean absorbance (*n* = 6) and viable bacterial colony recovery are expressed as log 10 cfu/mL (*n* = 3). (A, B) *A. hydrophila* strain 15. (C, D) *A. hydrophila* strain 97. (E, F) *E. tarda* NCIMB 2034. The error bars on the absorbance charts indicate the standard deviation of the mean. Disinfectant concentrations connected with the same letter subscripts are not significantly different within or across treatment groups. The threshold (red dotted line) for viable bacterial recovery represents the initial bacterial concentration (cfu/ml) added to the experiment.

#### 3.1.2 *Aeromonas hydrophila* strain 97

Similarly, OD values did not exceed 0.2 in any of the *A. hydrophila* strain 97 test groups at 0 h, nor in the 1% (dark blue), 0.5% (orange) and 0.25% (green) test groups after 24 h across all incubation temperatures (Figure 3C). In contrast, the OD value increased after 24 h in the 0.125% group (light blue) at both 28°C and 34°C (Figure 3C). Notably, the observed increase differed significantly from the OD value of the control group at 22°C and 28°C (*p* < 0.0001), while no significant difference was noted at 34°C (*p* = 1.000). Additionally, analysis of OD value changes during incubation (0–24 h) alongside viable bacterial recovery confirmed the bactericidal activity of the 1%, 0.5% and 0.25% test groups under all temperature conditions (Figure 3D). Nonetheless, viable bacterial growth was detected in the 0.125% group at every incubation temperature after 24 h. Further examination revealed that bacterial concentrations recovered from the 0.125% group were marginally higher than the initial concentration at 34°C, with an approximate 3‐log reduction recorded at 22°C (Figure 3D). These findings suggest diminished efficacy compared to *A. hydrophila* strain 15, for which a greater than 5‐log reduction was achieved in the same 0.125% group at 22°C.

#### 3.1.3 *Edwardsiella tarda* NCIMB 2034

For *E. tarda* NCIMB 2034, the OD of 1% test group (dark blue) at 0 h was slightly above 0.1 and less than 0.05 for the other test groups across test temperatures (Figure 3E). Consistent with observations in *A. hydrophila* strains, post‐incubation OD values for both the control group (purple) and the 0.125% (light blue) *E. tarda* group displayed exponential increases corresponding with the rise in incubation temperature (Figure 3E). However, no significant differences were detected between the OD values of both groups after 24 h at any temperature evaluated (*p* = 0.5337, 0.4027 and 0.9545, respectively), indicating similar bacterial proliferation rates in both the 0.125% and control groups. In contrast to the results obtained for *A. hydrophila* , the post‐incubation OD readings for the 0.5% (orange) and 0.25% (green) *E. tarda* groups rose to 0.07 at 28°C and 0.1 at 34°C, respectively. Conversely, the 1% group exhibited a decrease in OD at both temperatures (Figure 3E).

Furthermore, OD changes at concentrations of 0.5% or 0.25% did not yield viable bacterial growth, indicating that residual debris from lysed bacterial cells may have affected OD measurements in these groups. Results further demonstrated recovery of viable bacterial colonies from the 0.125% group across all incubation temperatures after 24 h, as well as from the 0.25% group at both 22°C and 34°C. Notably, recoveries from the 0.25% group at both temperatures exceeded a 5‐log reduction relative to the initial bacterial concentration introduced (Figure 3F). These findings suggest diminished efficacy of the disinfectant against *E. tarda* at 22°C and 34°C, in contrast to performance against *A. hydrophila* strains. Additionally, at 22°C, a 2–log decrease from the initial bacterial concentration was observed in the control group, indicating that bacterial proliferation was affected under this temperature condition (Figure 3F).

#### 3.1.4 *Streptococcus agalactiae* strain 6

For *S. agalactiae* strain 6, initial OD readings at 0 h were below 0.2 in the 1% group (dark blue) and under 0.05 in other test groups at various incubation temperatures (Figure 4A). After 24 h, elevated OD values were evident in the 0.5% (orange), 0.25% (green) and 0.125% (light blue) groups at both 28°C and 34°C (Figure 4A). Notably, the 1% group displayed variable OD values after 24 h, especially at 0 h for 22°C and 34°C. However, these variations did not align with viable bacterial recovery. This observation suggests that increased OD following incubation may result from turbidity associated with nonviable bacterial cells or a combination of factors, since no viable colonies emerged from these samples (Figure 4B). Moreover, viable bacterial counts in the control group supported the absorbance data, with colony growth observed across all tested temperatures. Further analysis indicated 5–log and 1–log reductions at 22°C and 34°C, respectively, along with a 2–log increase at 28°C compared to the initial bacterial concentration (Figure 4B). Collectively, these findings imply that temperature may have affected the organism's growth dynamics.

**FIGURE 4:**
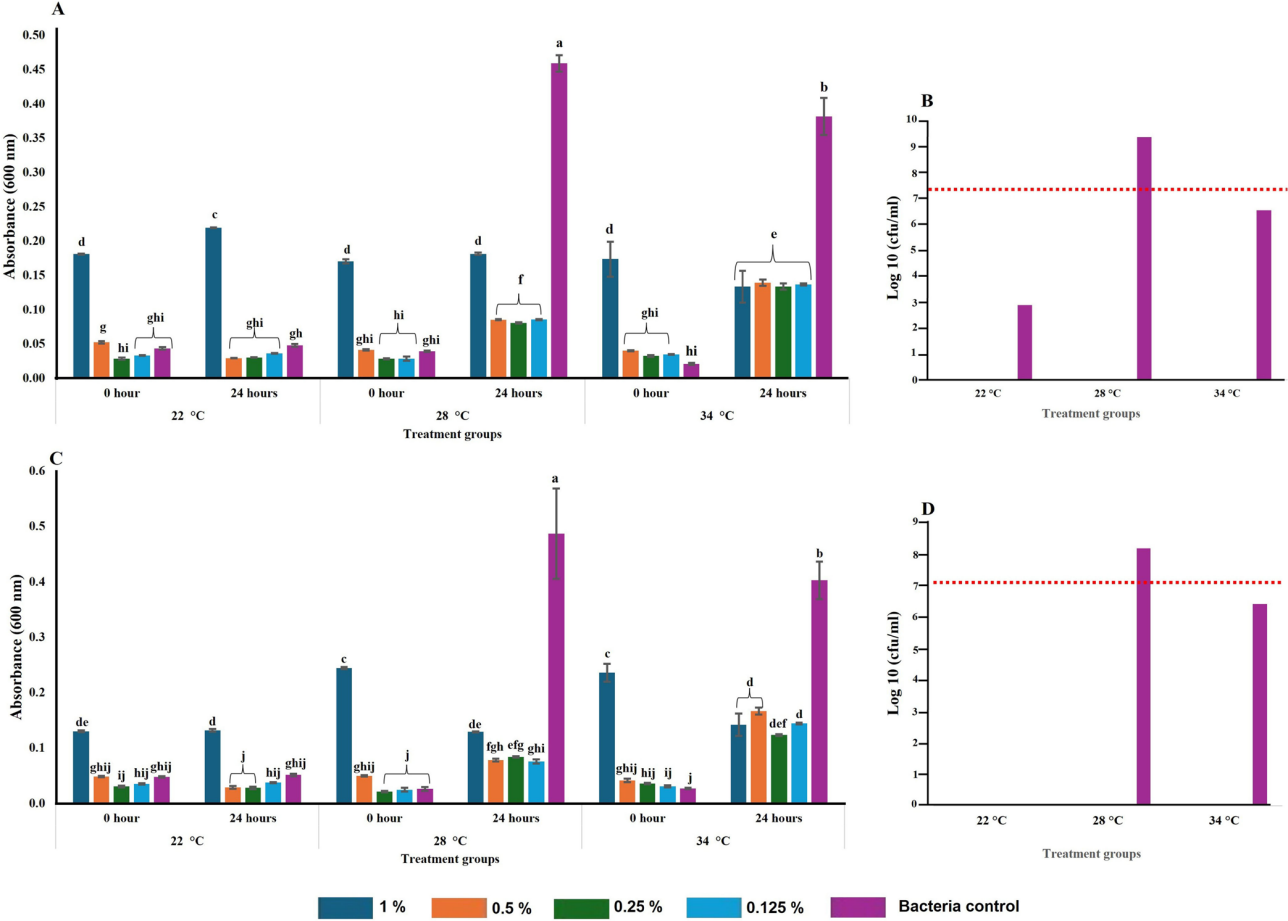
Absorbance and viable colony count of *S. agalactiae* isolates. The mean absorbance (*n* = 6) and viable bacterial colony recovery are expressed as log 10 cfu/mL (*n* = 3). (A, B) *S. agalactiae* strain 6. (C, D) *S. agalactiae* NCIMB 71348. The error bars on the absorbance charts indicate the standard deviation of the mean. Disinfectant concentrations connected with the same letter subscripts are not significantly different within or across treatment groups. The threshold (red dotted line) for viable bacterial recovery represents the initial bacterial concentration (cfu/mL) added to the experiment.

#### 3.1.5 *Streptococcus agalactiae* NCIMB 701348

The suspension test results for *S. agalactiae* strains highlighted an improved disinfection efficacy and clearly deviated from the patterns observed for both the *A. hydrophila* and *E. tarda* strains. Although the two *S. agalactiae* strains behaved similarly, our data revealed that some differences existed between them. For example, the absorbance data for *S. agalactiae* NCIMB 701348 revealed an increase in the OD after 24 h at 28°C and 34°C, with values that differed significantly (*p* < 0.0001) (Figure 4C). Second, no viable colonies were recovered from the *S. agalactiae* NCIMB 701348 control group at 22°C. In contrast, the number recovered both at 28°C and 34°C was comparable to those of *S. agalactiae* strain 6. However, their concentrations differed by ±1–log from the initial bacterial concentration (Figure 4D). Additionally, the viable growth in *S. agalactiae* NCIMB 701348 aligned with the absorbance data, similar to those of *S. agalactiae* 6. Consequently, no viable colonies were recovered in the test groups after 24 h of incubation, despite the change in OD recorded in the 0.5%, 0.25% and 0.125% groups at 28°C (Figure 4C,D). These results indicate enhanced efficacy of the tested disinfectant against *S. agalactiae* strains.

In summary, the broth microdilution suspension assay revealed a significant increase in the absorbance of all the tested isolates after 24 h incubation at 28°C and 34°C. Remarkably, not every change in absorbance following incubation was linear to viable bacterial growth. Above all, the inherent colour of the disinfectant further contributed to turbidity, resulting in significantly higher absorbance in the 1% test group at 0 h compared to other groups.

### 3.2 Carrier Tests

The carrier test assays imitate disinfection practices with nets and were conducted to evaluate the potential risk of poor disinfection with nets that are used in aquaculture facilities. The positivity of the assays was determined by an increase or decrease in optical density (OD) after 24 h of incubation, and the results revealed variability in disinfection efficacy among the tested bacterial strains.

#### 3.2.1 *Aeromonas hydrophila* strain 15

Carrier tests performed with *A. hydrophila* strain 15 revealed variable post‐incubation OD values in comparison with the initial OD. Particularly, the 1% (dark blue) and 0.5% (red) groups had OD values below 0.2, while the 0.25% (yellow) and 0.125% (purple) groups had readings above 1.2 across the temperatures (Figure 5A). However, OD values recorded in the 1% and 0.5% groups were barely visible on the chart, and no bacterial colonies were recovered in these groups (Figure 5A). Distinctly, both the 0.25% and 0.125% groups displayed poor disinfection since viable bacteria were found in both under tested temperature conditions (Figure 5A). These outcomes highlighted a shift from observations in the suspension test with the same isolate.

**FIGURE 5:**
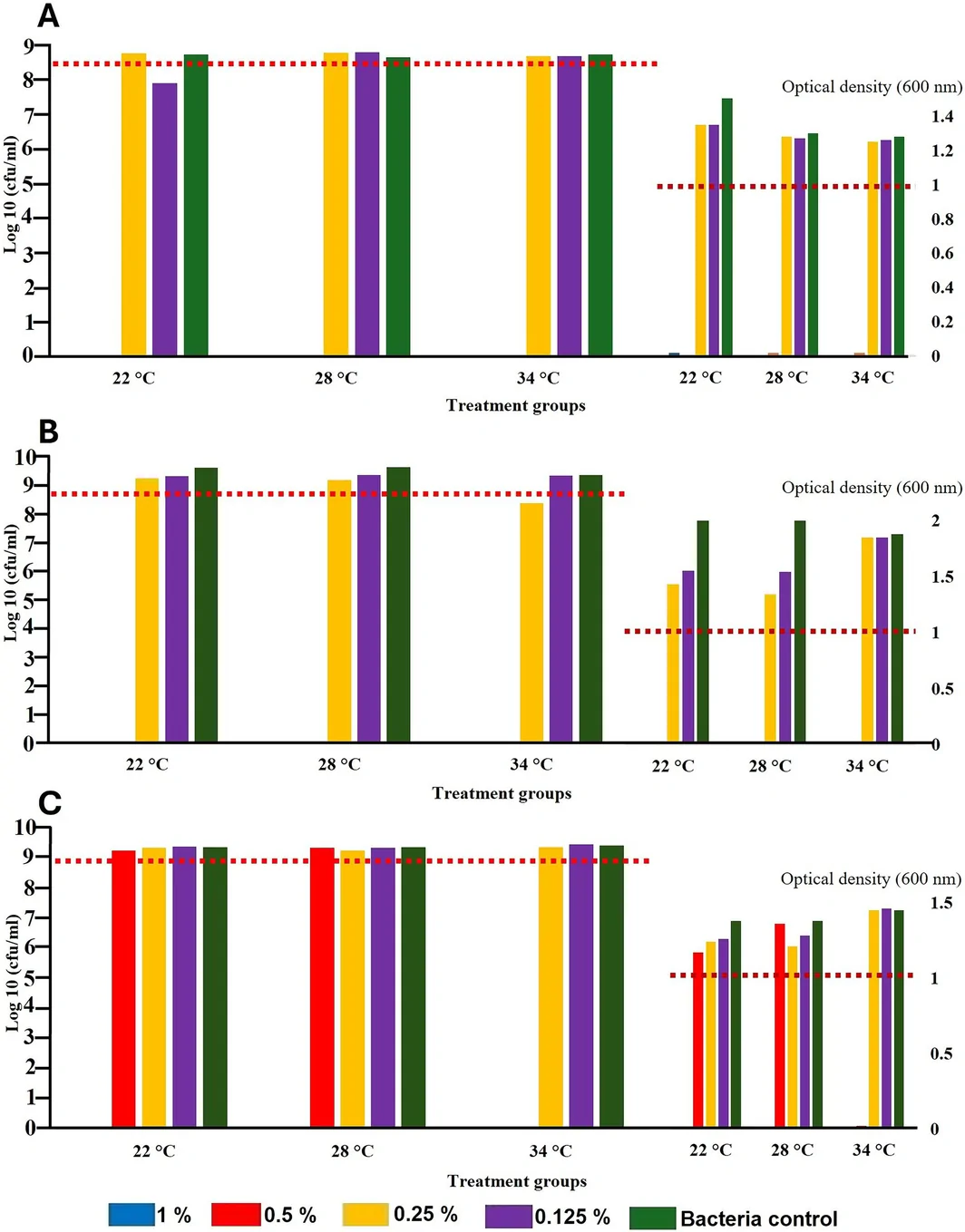
Viable colony count and cell density of *A. hydrophila* and *E. tarda* isolates. The viable bacterial colony recovered is expressed as log 10 cfu/mL (*n* = 3) (primary axis) and optical density (*n* = 2) (secondary axis). (A) *A. hydrophila* strain 15. (B) *A. hydrophila* strain 97. (C) *E. tarda* NCIMB 2034. The thresholds (dotted red line) represent the initial concentration (cfu/mL) on the primary axis and the initial cell density on the primary and secondary axes, respectively.

Additionally, post‐incubation recoveries in the 0.25% group were comparable in concentration to the initial bacterial concentration across all tested temperatures, while the 0.125% group exhibited a 1–log reduction at 22°C (Figure 5A). Unexpectedly, disinfection was less effective with the 0.125% at 22°C compared to the 0.25%, despite similar OD values. The reason for this disparity is unclear since repeated assays confirmed the result. Although the log reduction in the 0.125% group at 22°C was not significant, we suspect that unpredictable and multifactorial influences may have contributed to the outcome.

#### 3.2.2 *Aeromonas hydrophila* strain 97

As observed in the carrier test with *A. hydrophila* strain 15, post‐incubation OD values exceeded the initial measurement in the *A. hydrophila* strain 97 groups at concentrations of 0.25% (yellow) and 0.125% (purple) groups. Meanwhile, the control groups (green) exhibited a twofold increase across all tested temperatures (Figure 5B). In contrast to the *A. hydrophila* strain 15, no OD readings were detected in either the 1% or 0.5% groups of the *A. hydrophila* 97 across all incubation temperatures (Figure 5B). At 34°C, the OD values for the control (green), 0.25% and 0.125% groups were similar but differed at other temperatures. Additionally, the post‐incubation increase in OD observed in the control, as well as in the 0.25% and 0.125% groups, correlated with viable bacterial recovery at all temperatures (Figure 5B). The results further revealed that the concentration of bacteria recovered from the 0.25% closely matched that of initial concentration, except for slight differences in counts at 34°C (Figure 5B). Moreover, the viable bacterial concentration in the 0.125% group increased from 10^8^ to 10^9^ as temperature increased. Notably, no viable bacteria were detected in the 1% or 0.5% groups at any incubation temperature (Figure 5B).

#### 3.2.3 *Edwardsiella tarda* NCIMB 2034

Carrier test with *E. tarda* NCIMB 2034 showed that at 34°C, the control (green), 0.25% (yellow) and 0.125% (purple) groups exhibited comparable OD values, whereas differences were observed at other temperatures (Figure 5C). All OD readings remained below 1.5, unlike in the *A. hydrophila* strain 97, where it exceeded 1.5. For the *E. tarda* strain, OD values in the 0.5% (red) group stayed below 1.5 both at 22°C and 28°C (Figure 5C). Notably, all post‐incubation OD measurements exceeded the initial OD except for the 0.5% group at 34°C, which was not visible on the chart (Figure 5C). Additionally, the data revealed a positive correlation between post‐incubation OD and viable bacterial recovery in the bacteria control, 0.5%, 0.25% and 0.125% groups across all tested temperatures (Figure 5C). These outcomes highlight poor disinfection with the 0.5% concentration both at 22°C and 28°C, since viable colonies were recovered from the sample, in contrast to the *A. hydrophila* strains (Figure 5C). Remarkably, the number of bacteria recovered in both bacteria control and test groups matched the initial bacteria concentration introduced in each assay at all temperatures (Figure 5C). Whereas no viable bacteria were detected in the 1% group at any temperature or in the 0.5% group at 34°C (Figure 5C).

#### 3.2.4 *Streptococcus. agalactiae* strain 6

In the *S. agalactiae* strain 6 carrier tests, OD increase that surpassed the initial OD was observed in the bacteria control groups (green) at all temperatures evaluated (Figure 6A). Conversely, OD readings remained below 0.2 for the 1% (dark blue) at 22°C, 0.5% (red) at 28°C and across all test groups at 34°C, although these data points were not very visible on the chart (Figure 6A). Additionally, viable bacterial recovery correlated to post‐incubation OD records exclusively within the bacteria control groups, where recovered concentrations matched those of starting concentration (Figure 6A). No bacterial proliferation was observed in any of the test groups across the temperatures, demonstrating enhanced disinfection efficacy against *S. agalactiae* strain 6 (Figure 6A). The results from the *S. agalactiae* strain 6 carrier tests aligned with data from the suspension tests particularly among the test groups but differed from the bacteria control groups and the carrier test outcomes for both *A. hydrophila* and *E. tarda* strains.

**FIGURE 6:**
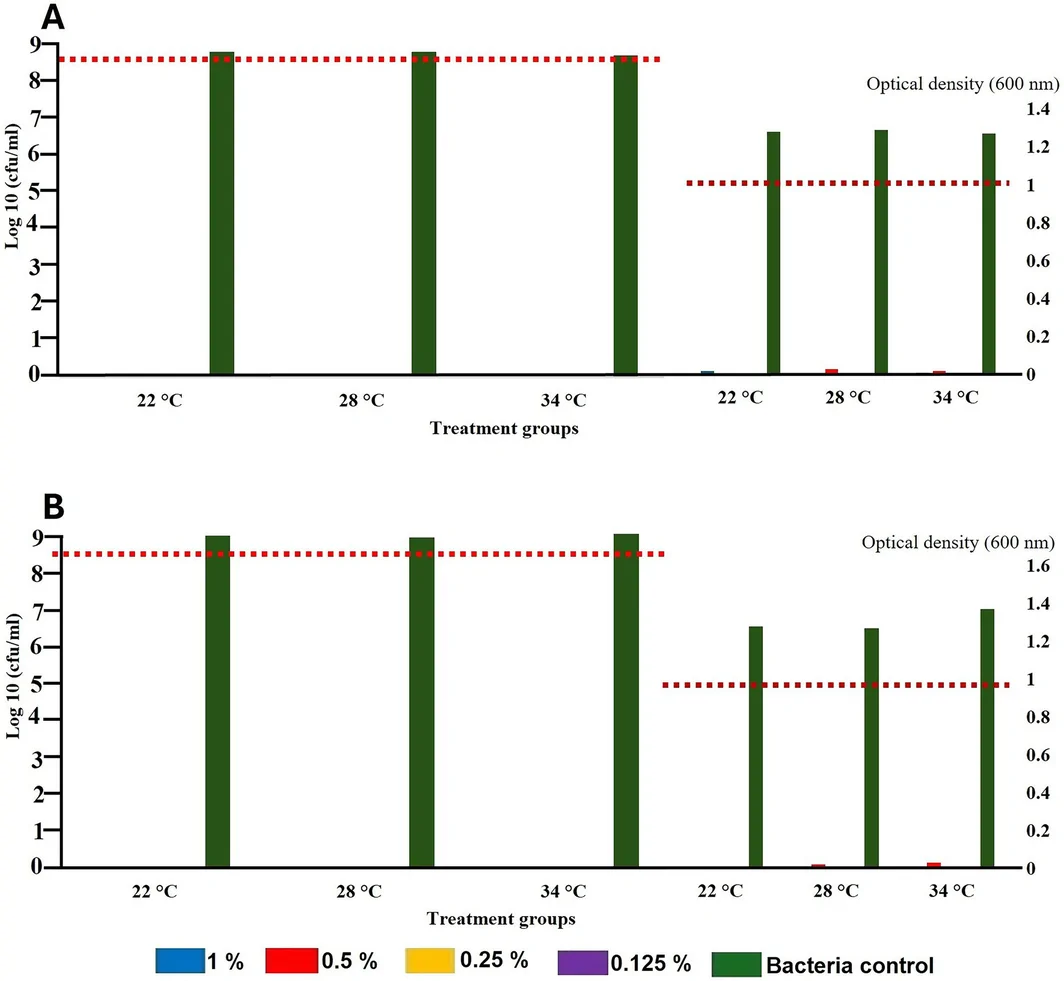
Viable colony count and cell density measurement of *S. agalactiae* isolates. The viable bacterial colony recovered is expressed as log 10 cfu/mL (*n* = 3) (primary axis) and optical density (*n* = 2) (secondary axis). (A) *S. agalactiae* strain 6. (B) *S. agalactiae* NCIMB 71348. The thresholds (dotted red line) represent the initial concentration (cfu/mL) on the primary axis and the initial cell density on the primary and secondary axes, respectively.

#### 3.2.5 *Streptococcus agalactiae* NCIMB 701348

For the *S. agalactiae* NCIMB 701348 carrier test, post‐incubation OD value exceeded 1.2 in the bacteria control (green) across the temperatures. Whereas it was less than 0.2 in the 0.5% test group (red) at 28°C and 34°C (Figure 6B). Additionally, the viable bacterial counts in the bacteria control groups were consistent across the temperatures evaluated and closely matched the starting concentrations (Figure 6B). Again, as with *S. agalactiae* strain 6, the bacterial counts in the bacteria control groups showed less than a 1‐log difference from the initial concentration, whereas no viable bacteria were detected in all the test groups (Figure 6B).

In addition, a comparison of the suspension and carrier test results revealed differing outcomes, particularly for *A. hydrophila* and *E. tarda* strains. In the suspension tests, *A. hydrophila* strains grew only in the presence of 0.125% disinfectant (Figure 3B,D), whereas in the carrier tests, both the 0.25% and 0.125% test groups exhibited viable growth at all temperatures (Figure 5A,B). Moreover, the variability was greater for *E. tarda* ; for example, at 22°C and 28°C, carrier tests recorded viable growth up to the 0.5% test group (Figure 5C). Whereas in the suspension test, proliferation of *E. tarda* was pronounced at lower disinfectant concentrations across all temperatures (Figure 3F). However, the log reductions in the 0.25% group at 22°C and 34°C in the suspension tests remained below 5–log compared with those in the *E. tarda* control group.

Furthermore, variability was not obvious in either of the tests conducted with *S. agalactiae* strains, as no viable growth was recorded under the experimental conditions examined. Nevertheless, the outcomes differed slightly in the *S. agalactiae* NCIMB 701348, as this strain exhibited viable growth in the carrier test control group at 22°C (Figure 6B), whereas no growth was observed in the suspension test (Figure 4D).

### 3.3 MIC and MBC for the Isolates

The distinctions in the outcomes of the two tests were apparent in both the MIC and MBC records for the isolates (Table 4). At 22°C, all bacterial isolates had an MIC of 0.125% in both tests, with *S. agalactiae* isolates exhibiting identical MIC and MBC values under all tested conditions (Table 4). In contrast, at higher incubation temperatures of 28°C and 34°C, the MIC increased to 0.25% for *A. hydrophila* and *E. tarda* strains, indicating reduced efficacy of the disinfectant. Notably, the MICs for these strains consistently rose with temperature increase. Higher temperatures resulted in a decreased MBC for *A. hydrophila* in the suspension test, whereas MBC for *E. tarda* displayed variability without a distinct trend (Table 4). In the carrier test, the MBC for *A. hydrophila* stayed the same regardless of temperature, while *E. tarda* 's MBC value dropped at the highest incubation temperature (Table 4). Collectively, these data suggest that temperature exerts a considerable influence on both MIC and MBC for these organisms.

**TABLE 4:** MIC and MBC results for the suspension and carrier tests.

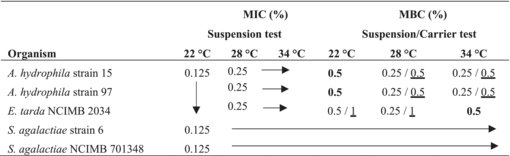

*Note:* Values in bold represent MBC for both suspension and carrier tests under the same incubation temperature, underlined values are MBC for carrier tests, and arrows designate same MIC/MBC values.

## 4 Discussion

A substantial number of bacterial organisms are pathogenic to aquatic species like catfish (Dayana Senthamarai et al. 2023; Irshath et al. 2023). Consequently, the bacterial strains investigated in this study are representative of aquatic bacterial pathogens relevant to catfish and tilapia aquaculture worldwide. The selected isolates consisted organisms of varying phenotypic characteristics and distinct virulence factors that are commonly associated with aquatic pathogens (Colussi et al. 2022; Semwal et al. 2023; Xu and Zhang 2014). Use of Virkon aquatic disinfectant in aquaculture facilities is widespread across many countries due to its proven effectiveness against a broad spectrum of aquatic pathogens (Stockton‐Fiti and Moffitt 2017; West et al. 2018). According to the manufacturer, Virkon aquatic disinfectant has a minimal environmental impact. It degrades quickly, remains effective against numerous pathogens even at temperatures as low as 4°C or higher than 22°C, and stays stable in solution for over 7 days (Stockton‐Fiti and Moffitt 2017). In this study, its effectiveness was evaluated following European Standards for chemical disinfectants and antiseptics, which stipulates at least a 5‐log reduction in bacterial counts after treatment as a measure of efficacy (Mon‐on et al. 2018). To ensure that sufficient bacterial cells were exposed to the disinfectant, high bacterial concentrations were used. Additionally, pilot studies revealed that *S. agalactiae* strains grow back more slowly after treatment with the disinfectant compared to *A. hydrophila* and *E. tarda* isolates. Hence, the post‐treatment bacterial recovery times were 43 h for *S. agalactiae* and 28 h for both the *A. hydrophila* and *E. tarda* strains.

Two laboratory‐based methods that are routinely applied when investigating disinfection efficacy were employed. The broth microdilution method, also known as the suspension test, allows direct interaction between disinfectants and test organisms in small‐scale assays (Horváth et al. 2016) and is often used to establish the minimum inhibitory concentration (MIC) of antimicrobial agents (CLSI 2018). Conversely, the carrier method, or carrier test, is considered more appropriate for evaluating antimicrobial efficacy in aquaculture settings (Jang et al. 2017); it involves assays in larger volumes where organisms come into contact with disinfectants via a carrier. This approach better imitates real‐world farming scenarios, where pathogens are directly exposed to disinfectants.

Suspension tests, however, often result in bacterial cells attaching to the surfaces of experimental materials, making complete recovery and enumeration difficult (Jang et al. 2017). Despite twice washing of the experimental wells, residual bacterial cells were detected upon assessment of their sterility status. Furthermore, our data showed that the two methods did not align with each other under any of the tested conditions. For example, the suspension test with *A. hydrophila* strain 15 required a lower disinfectant concentration for complete inactivation compared to the carrier test, which required a higher concentration to achieve the same result. Additionally, MBC of *A. hydrophila* strains in the suspension tests varied with temperature but remained consistent in the carrier test regardless of temperature. This disparity was more pronounced in experiments performed with *E. tarda* , which was completely inactivated with a low disinfectant concentration in the suspension test, but required higher concentrations in the carrier tests. These findings corroborate previous findings emphasizing that the carrier test offers a more reliable measure of efficacy of chemical disinfectants under real‐world conditions such as aquaculture systems (Jang et al. 2017). The study conducted by Jang et al. (2017) compared two types of carriers with different surface structures—birch wood pieces and stainless steel. In contrast, our current research simulated an actual aquaculture setting by using nets, which are common in aquaculture facilities. Despite these methodological differences, both studies demonstrate that employing carriers offers a more realistic approach than suspension methods when assessing the effectiveness of chemical disinfectants in aquaculture.

Nevertheless, disinfection efficacy against bacterial pathogens is influenced by several intrinsic and extrinsic factors, with environmental temperature having a greater impact. Recent studies link rising water temperatures from climate change to higher disease incidences and increased stress in fish species (Okon et al. 2024). Temperature variation impacts both disinfection efficacy and bacterial growth rates, with higher temperatures potentially improving the disinfection efficacy of disinfectants (Mon‐on et al. 2018). However, this relationship is not linear because extreme temperatures can compromise the interactions and mechanisms of action of chemical disinfectants, which are temperature dependent (Jang et al. 2017). For example, chemical disinfectants, including Virkon aquatic disinfectants, employ oxidation reactions to weaken sulphur bonds in the proteins and enzymes of target organisms, resulting in eventual inactivation (Acosta et al. 2021). Disinfection is essential in aquaculture for maintaining clean culture systems and sanitizing equipment throughout production cycles (Bowker et al. 2019; Mohamed Din and Subasinghe 2024). Our data revealed notable differences in susceptibility patterns, particularly between the two gram‐negative bacteria. The *E. tarda* strain tolerated broader Virkon aquatic concentration ranges than the *A. hydrophila* strains did, and the concentration required for complete inactivation varied between both species. However, an increase in temperature improved the disinfectant's efficacy against *E. tarda* , indicating that changes in temperature can significantly impact the disinfection efficacy of chemical disinfectants (Dvorak 2023; Mon‐on et al. 2018). This result is consistent with the findings of Mon‐on et al. (2018) who observed a 5‐log reduction at 30°C following treatment of two strains of *S. agalactiae* with Anidine 100, BKC 80%, and Chloraldehyde. Additionally, their study highlighted that lower temperatures diminished the disinfection efficacy of both Anidine 100 and Chloraldehyde. Furthermore, Jang et al. (2017) found that *S. typhimurium* could be inactivated within 5 min at 25°C using six different disinfectants and suggested that extending the contact time would further enhance the effectiveness of the disinfectants. This suggests that reduced temperature conditions may impact how well disinfectants work. Although the present study was not field based, the data embody relevant information to support disinfection practices as part of biosecurity measures in aquaculture systems. However, our data showed that temperature strongly impacted lower concentrations primarily due to its effects on reaction rates, molecular interactions and cellular damage (Maillard and Pascoe 2024; Moon et al. 2023; Peleg 2021). At low disinfectant levels, these factors limit effectiveness, but higher temperatures enhance bactericidal activity. In contrast, when disinfectant concentrations are high, the system operates near optimal microbial killing capacity, so increasing temperature has minimal benefit.

Naturally, bacterial species possess different innate properties that support their survival and reproduction even in hostile environments (Tong et al. 2021). Thus, the differences in inactivation patterns among the bacterial species in this study could be a consequence of their differing innate properties (Malvestiti and Dantas 2019). Notably, gram‐negative bacterial species, including *A. hydrophila* and *E. tarda*, possess an outer membrane enriched with LPS that provides extra protection against chemical attacks (VanOtterloo and Trent 2024). Compared with those in gram‐positive bacteria, the stable layers of LPS in gram‐negative bacterial species result in lower permeability, enabling greater resistance to toxic chemicals (Yeow et al. 2023). This characteristic may have contributed to the reduced disinfection efficacy observed in gram‐negative strains, which require higher disinfectant concentrations for inactivation than those needed for gram‐positive *S. agalactiae* strains. Moreover, differences in virulence factors can lead to varying intrinsic resistance mechanisms to disinfectants among microorganisms (Tong et al. 2021). *E. tarda* produces siderophores, which are small‐molecule metal chelators that support the ability of bacteria to absorb iron from their environment, including host proteins, especially when iron scarcity occurs (Albelda‐Berenguer et al. 2019). These siderophores are characterized by iron acquisition properties (Kramer et al. 2020) but are also known to contribute to disinfectant resistance in bacterial species, specifically by reducing the effectiveness of reactive oxygen stress (ROS)‐based disinfectants (Arnold 2024). Previous studies show that a lack of enterobactin, a siderophore, increased the susceptibility of *Escherichia coli* to oxidative damage from hydrogen peroxide and paraquat, whereas pyoverdine enhanced resistance to ROS‐mediated killing in *P. aeruginosa* (Jin et al. 2018; Peralta et al. 2016). Additionally, the cell wall of *E. tarda* contains OMPs, which include transmembrane proteins and lipoproteins that provide additional protection against assault on bacterial cells (Otzen and Andersen 2013; Yeow et al. 2023). Collectively, these findings indicate that the combination of innate properties in *E. tarda* may have contributed to its high tolerance to the disinfectant compared to other organisms evaluated in this study.

Furthermore, the killing of *S. agalactiae* by the disinfectant at the lowest concentration could be due to its nonmotile nature. Although motility does not influence disinfectant mechanisms, such as disruption of the cell membrane, protein or DNA (McDonnell and Russell 1999). However, it can alter the exposure time, as motile bacteria can potentially move away from a localized environment with high disinfectant concentrations (Piskovsky and Oliveira 2023). Although the influence of bacterial motility on the efficacy of chemical disinfectants has not been fully explored, motile organisms can more easily escape from uncomfortable environments than nonmotile organisms. Additionally, most motile bacterial species form protective layers known as biofilms, which increase their resistance to disinfectant penetration and consequently reduce the overall effectiveness of disinfectants (Zhao et al. 2023). Our findings indicated that a high disinfectant concentration was required for full inactivation of gram‐negative motile organisms, which agreed with earlier studies showing that *Vibrio harveyi* and *V. anguillarum* were inactivated with similar concentration of Virkon aquatic disinfectant (Acosta et al. 2021). This supports the idea that virulence factors play significant role in how microbes resist disinfection agents.

The intrinsic nature of bacterial species can influence their behaviour and response to disinfectants. For example, *S. agalactiae* strains produce polysaccharide capsules that contain sialic acid (Liu and Liu 2022), a virulence factor that supports pathogenesis and defends bacterial cells from complement deposition and phagocytosis (Dudek et al. 2022; Engibarov et al. 2025). At present, it is not yet fully understood whether capsule formation interferes with the disinfection efficacy of chemical disinfectants against bacterial pathogens. However, the capsule type influenced the resistance pattern of *Acinetobacter baumannii* strains to benzethonium chloride, benzalkonium chloride and chlorhexidine gluconate (Tipton et al. 2018). Whereas in *A. baumannii* AB5075, capsule production did not significantly influence the activity of benzalkonium chloride. Both studies employed two different methods: the CFU enumeration technique, which is specific for revealing marginal differences in antimicrobial resistance (Tipton et al. 2018), and broth microdilution assays (Nunez et al. 2023). Our present study combined both methods under variable temperature conditions and compared the data across multiple bacterial isolates with distinct characteristics. And the study revealed high efficacy of Virkon aquatic disinfectant against *S. agalactiae* , indicating that the pattern with which capsule production impact disinfection efficacy varies among gram‐positive bacteria.

Nonetheless, assays to detect capsule formation in *S. agalactiae* isolates were not incorporated into this study, nor was post‐treatment phenotypic characterization conducted to assess alterations in bacterial cells following exposure to the disinfectant. Scanning electron microscopy could serve as an effective method for verifying capsule production in *S. agalactiae* isolates and for identifying phenotypic modifications in cells recovered after treatment with Virkon aquatic disinfectant. Previous studies revealed that antimicrobial agents can alter the phenotypic properties of bacterial species, including altering their general morphology, cell surface, membrane and pump functions after treatment with antimicrobial agents (Chegene Lorestani et al. 2023; Cushnie et al. 2016). Hence, we suspected that capsule production contributed to the turbidity observed at higher disinfectant concentrations in suspension tests with *S. agalactiae* strains. Unlike in *A. hydrophila* and *E. tarda* , an increase in the post‐incubation absorbance of *S. agalactiae* strains did not result in viable colony recovery. Additionally, capsule production could have influenced the formation of visible cell granules, which also influenced the absorbance readings following *S. agalactiae* inactivation at high disinfectant concentrations.

In addition, the *S. agalactiae* strains in this study were slow‐glowing organisms requiring more hours for regrowth than strains of *A. hydrophila* and *E. tarda* . The slow growth of the *S. agalactiae* strains may have contributed to their quick inactivation at the lowest disinfectant concentration. Although studies have not fully explored the impact of bacterial growth rate on disinfection efficacy. Undoubtedly, fast‐growing bacterial organisms can easily produce millions of cells, thereby increasing their population as a survival strategy to counteract the killing effects of antimicrobial agents (Cherchi and Gu 2011). Furthermore, the mechanisms of disinfectant resistance within aquatic bacterial species are broad. In the presence of chemical disinfectants, bacterial species undergo a series of reactions to suppress invasion and lower disinfectant concentrations (Tong et al. 2021). They also activate the oxidative stress response, leading to the expression of elevated levels of reactive oxygen species that can impact the bactericidal properties of disinfectants (Li et al. 2017). Moreover, it is common for bacteria to trigger specific DNA damage responses for DNA repair and adapt to disinfectants (Lu et al. 2018). It is likely that the *A. hydrophila* and *E. tarda* strains in this study initiated specific intrinsic responses to resist the killing effects of Virkon aquatic disinfectant, thus increasing their tolerance levels.

The use of suboptimal disinfectant concentrations is a serious health concern and risk in aquaculture, considering the large spectrum of bacterial pathogens present in aquaculture environments with varying levels of sensitivity and tolerance to chemical disinfectants. Poor disinfection practices encourage persistent bacteria, leading to disinfectant resistance (Abd El‐Hack et al. 2022). Additionally, the use of sublethal disinfectant concentrations could increase the accumulation of bacterial cells in a viable but nonculturable state (Pazos‐Rojas et al. 2023), which may eventually regain viability within aquaculture production systems. Poor sanitation practices due to suboptimal disinfectant application among catfish farmers, especially in developing economies, are serious food safety and fish welfare concerns with enormous economic consequences. Such practices could induce biofilm formation and increase morbidity levels in aquaculture systems, leading to high mortality rates and eventual loss in investment. In addition, poor sanitation enhances regular disease outbreaks in production systems with a consequent need for additional treatments, which impacts cost of production and causes environmental damage. Collectively, these could have contributed to the setbacks that have persisted in the catfish aquaculture production sector, especially the stagnation of African catfish production in Nigeria (Ogunji and Wuertz 2023).

## 5 Conclusion

The findings from this study revealed that temperature variation, disinfectant concentration and virulence factors inherent in aquatic pathogens are the key factors influencing disinfection efficacy. Our findings further highlighted that carrier tests are ideal methods for evaluating the efficacy of disinfectants that are applied against aquatic pathogens, and that the application of suboptimal disinfectant concentrations is associated with health and safety concerns, as they could lead to the accumulation of pathogens in a viable but nonculturable state.

## Author Contributions

**Margaret Crumlish:** conceptualization, methodology, resources, supervision, writing – review and editing. **Aloysius Chimezie Adibe:** conceptualization, investigation, funding acquisition, writing – original draft, methodology, writing – review and editing, formal analysis, data curation.

## Funding

This study was supported by funds provided by the Tertiary Education Trust Fund of the Nigerian government.

## Ethics Statement

The study was approved by the Research Ethics Review Board of the University of Stirling, UK, with reference number EC20242154215596. The approved guidelines and regulations were strictly followed to conduct all the experiments.

## Consent

The authors have nothing to report.

## Conflicts of Interest

The authors declare no conflicts of interest.

## Data Availability

All the data and materials are embedded within the body of the article.
